# Evaluation of a Newly Developed Vacuum Dried Microtiter Plate for Rapid Biocide Susceptibility Testing of Clinical *Enterococcus faecium* Isolates

**DOI:** 10.3390/microorganisms8040551

**Published:** 2020-04-11

**Authors:** Alice Roedel, Ralf Dieckmann, Oliwia Makarewicz, Anita Hartung, Matthias Noll, Mathias W. Pletz, Sascha Al Dahouk, Szilvia Vincze

**Affiliations:** 1 German Federal Institute for Risk Assessment, Department of Biological Safety, 10589 Berlin, Germany; alice_roedel@gmx.de (A.R.); sascha.al-dahouk@gmx.de (S.A.D.); 2 Institute for Infectious Diseases and Infection Control, Jena University Hospital, 07747 Jena, Germany; oliwia.makarewicz@med.uni-jena.de (O.M.); anita.hartung@med.uni-jena.de (A.H.); mathias.pletz@med.uni-jena.de (M.W.P.); 3 Research Campus Infectognostics, 07743 Jena, Germany; 4 Institute for Bioanalysis, University of Applied Sciences and Arts, 96450 Coburg, Germany; matthias.noll@hs-coburg.de; 5 Department of Internal Medicine, RWTH Aachen University Hospital, 52074 Aachen, Germany

**Keywords:** biocide susceptibility, *Enterococcus faecium*, vancomycin-resistant, VRE

## Abstract

We investigated the suitability of a newly developed biocide susceptibility test system based on microtiter plates containing vacuum dried biocides as a fast and reliable screening method. The evaluated substances included the cationic biocides benzalkonium chloride (BAC), chlorhexidine dihydrochloride (CHX), cetylpyridinium chloride, didecyldimethylammonium chloride, and octenidine dihydrochloride. Testing a selection of *Escherichia coli* and enterococci, the biocide microtiter plates provided results comparable to those obtained from broth microdilution according to ISO 20776-1. Broad MIC ranges allowed for testing gram-positive and gram-negative species with the same plate design. In the second part of our study, we applied the established method to analyze the susceptibility of 90 clinical *Enterococcus faecium* isolates from a German university hospital, as previous studies have indicated a link between reduced susceptibility to substances such as CHX and BAC and vancomycin resistance. We therefore determined MIC and minimum bactericidal concentrations (MBC) for 48 non-clonal vancomycin susceptible and 42 non-clonal vancomycin resistant isolates, but MIC_95_ and MBC_95_ were quite similar in both groups. Our easy to handle and ready to use test system enables the routine surveillance of bacterial tolerance towards disinfectants in hospitals. As a result, hygiene measures can be adapted and nosocomial infections controlled despite increasing prevalence of antibiotic-resistant bacteria.

## 1. Introduction

In the era of multidrug resistance with a rising number of infections unresponsive to antibiotic treatment, the relevance of hygiene measures to reduce bacterial burden and transmission in clinical settings has significantly increased [1]. Most disinfection strategies make use of a mixture of biocides with bacteriostatic and/or bactericidal activities simultaneously affecting different bacterial target sites [2]. The multifactorial mode of action led to the assumption that tolerance development in bacteria is rather unlikely. Nonetheless, numerous in vitro studies demonstrated the adaptation capability of various bacterial species when exposed to different biocidal substances in sublethal concentrations [3,4]. In addition, recent epidemiological studies provide support that decreasing biocide susceptibility can be caused by the introduction of biocides into the clinical environment. Pidot et al., for example, showed that *Enterococcus faecium* isolates obtained from two major hospitals in Melbourne, Australia after 2010 were 10-fold more tolerant to isopropanol compared to former isolates [5]. Decreasing susceptibility of *Staphylococcus aureus* to biocides such as chlorhexidine (CHX) and octenidine (OCT) over time was observed after increased usage of both substances in hospitals [6]. In several outbreak investigations, we observed reduced susceptibility to biocides of the predominantly used substances in outbreak isolates. We have recently described a clonal cluster of carbapenem-resistant *Klebsiella pneumoniae* isolates with decreased susceptibility to CHX [7]. These isolates were detected by regular screening of intensive care unit (ICU) patients on a ward that has implemented routine washing with CHX to decrease the rate of catheter-related infections. As a matter of concern, reduced CHX susceptibility was associated with resistance to colistin, likely caused by increased efflux of both substances via the same route. Furthermore, we have reported a polyclonal outbreak with *Serratia marcescens* on a neonatology ICU [8]. These isolates exhibited resistance to 0.5% Mikrobac forte^®^ consisting of benzyl-C12-18-alkyl dimethyl ammonium chloride 199 mg/g and *N*-(3-aminopropyl)-*N*-dodecylpropane-1,3-diamine 50 mg/g, which was used for disinfection of surfaces before the outbreak. One of the measures in the bundle that finally successfully contained this outbreak consisted of increasing the concentration of used Mikrobac forte^®^ to 2%. Outcomes of these studies point towards the need for regular monitoring of bacterial biocide susceptibility profiles. For this purpose, reliable high-throughput screening methods are needed that can be easily compared across studies. In contrast to antibiotic susceptibility testing, standardized procedures are missing for biocide susceptibility testing although attempts have been made to introduce respective protocols [9,10]. The need to harmonize biocide susceptibility testing methods was emphasized by a study highlighting the effect of slight modifications in the test procedure, such as choice of nutrient broth or assay plate material, on the results obtained [11]. Currently, biocide susceptibility is frequently tested by broth microdilution as it resembles the standardized procedure for antibiotic resistance testing according to ISO standard 20776-1 [12]. The method includes the fresh preparation of biocide stock solutions, which are diluted to a range of concentrations covering MICs and minimum bactericidal concentrations (MBCs). A defined number of bacterial cells (2 × 10^5^ – 8 × 10^5^ cfu/mL) is subsequently exposed to biocides for 18 ± 2 h at 34–37 °C. Concentration ranges for each substance are covered in doubling dilutions. The MIC is defined as the lowest concentration leading to inhibition of bacterial growth, which is determined visually or by measuring optical density. The aforementioned method is time-consuming and error-prone as ranges of biocidal substances need to be prepared prior to each investigation. Hence, a test plate system containing predefined concentrations of biocidal substances would be preferable for routine screening of bacterial susceptibility profiles to biocides of interest. Thus, the first aim of our study was to evaluate the comparability of susceptibility profiles for chosen biocides obtained with broth microdilution method according to ISO 20776-1 and a newly developed microtiter-plate containing vacuum dried cationic biocides (MERLIN Diagnostika GmbH, Bornheim-Hersel, Germany). Subsequently, we used the novel test system to determine biocide susceptibility of vancomycin-resistant (VRE) and susceptible enterococci (VSE) as vancomycin resistance has been linked to reduced susceptibility to cationic biocides such as CHX and benzalkonium chloride (BAC) in the past [13,14].

## 2. Materials and Methods

### 2.1. Bacterial Strains and Culture Conditions

In total, 95 field isolates and two reference strains were analyzed comprising of *Enterococcus* spp. and *Escherichia coli*. All enterococci field isolates were sampled at the Jena University Hospital. To verify the suitability of the newly developed test system, an initial strain panel was tested, consisting of four *E. coli* isolates from stable surfaces of broiler fattening farms and the *E. coli* reference strain ATCC 25922, as well as three clinical isolates of *E. faecium,* one of *Enterococcus faecalis,* and the *E. faecalis* reference strain ATCC 29212. The actual strain panel studied included 42 non-clonal VRE and 48 non-clonal VSE isolates (among them the three pretested *E. faecium*) from blood cultures (*n* = 73), swabs (*n* = 7), urine (*n* = 6), and fecal samples (*n* = 4; Table S1). Isolates were stored in glycerol stocks. Prior to use they were grown on Mueller–Hinton (MH) agar (Thermo Fisher Diagnostics GmbH Microbiology, Wesel, Germany) overnight at 37 °C. 

### 2.2. Biocides

The cationic biocides BAC (Sigma Aldrich, Steinheim, Germany), chlorhexidine dihydrochloride (CHX; Sigma Aldrich), cetylpyridinium chloride (CTP; TCI, Eschborn, Germany), didecyldimethylammonium chloride (DDAC; Sigma Aldrich), and octenidine dihydrochloride (OCT; Alfa Aesar by Thermo Fisher, Kandel, Germany) were tested including the following concentrations in doubling dilution steps: 256 to 0.5 mg/L (BAC), 128 to 0.25 mg/L (CHX), 256 to 1 mg/L (CTP), 128 to 0.5 mg/L (DDAC), and 32 to 0.125 mg/L (OCT).

### 2.3. Biocide Susceptibility Testing

#### 2.3.1. MIC Determination by Wet Plate Procedure

MIC values were determined for the initial strain panel using a broth microdilution method in accordance with the Clinical and Laboratory Standards Institute (CLSI) guidelines [15] and ISO 20776-1 [12]. Testing was carried out as previously published [16] except for MH broth (Thermo Fisher Diagnostics GmbH Microbiology) used instead of tryptic soy broth. In compliance with EN 1276, standardized hard water was used to freshly prepare all stock solutions and to subsequently adjust the desired concentrations. The MIC was defined as the lowest biocide concentration completely inhibiting bacterial growth, which was determined after additional visual inspection (data not shown). Optical density was measured with a Mithras^2^ Multimode Reader (Berthold Technologies, Bad Wildbad, Germany). OD_595_ = 0.04 and 0.08 were considered as cut-off values for enterococci and *E. coli*, respectively. Experiments were repeated on three different days in three technical replicates per day.

#### 2.3.2. MIC and MBC Determination by Dried Plate Procedure

In parallel, susceptibility testing was performed using a broth microdilution method with customized microtiter plates containing vacuum dried biocides (MERLIN Diagnostika GmbH). Briefly, 100 µl MH broth containing approximately 5 × 10^5^ cfu/mL were added to each well and plates were incubated for 20 ± 2 h at 37 °C. OD_620_ was measured with a Multiscan EX microplate photometer (Thermo Scientific, Vantaa, Finland). MIC values were defined after additional visual inspection (data not shown). OD_620_ = 0.08 was considered as the cut-off value for both enterococci and *E. coli*. MICs of the initial strain panel were tested on three different days in three technical replicates per day. Subsequently, MIC testing was conducted for the second strain panel in three biological replicates on three different days using the customized biocide microtiter plate. In addition, MBC tests were performed as previously described [16]. Dey–Engley neutralizing broth (Sigma Aldrich) was used to quench biocidal effects for MBC testing. The MBC was defined as the lowest concentration of the biocide that revealed no visible colonies on MH agar.

### 2.4. Comparative Analysis of Both MIC Testing Methods

Comparability of results obtained with both methods was assessed by applying two criteria of ISO 20776-2, essential agreement (EA), and reproducibility [17]. According to ISO 20776-2, alternative antimicrobial susceptibility testing methods need to be compared to the reference method for antibiotic resistance testing based on ISO 20776-1. So far, no reference method is available for biocide susceptibility testing. Hence, we compared the results of the MIC testing conducted with customized microtiter plates (dried plate procedure) with data obtained with broth microdilution according to ISO 20776-1 (wet plate procedure). The EA was calculated using the following formula:

EA = N_EA_ × 100/N

where N_EA_ = Number of isolates showing a modal value comparable to the modal value obtained with the ISO 20776-1 method (± one doubling dilution step), and N = Total number of tested isolates. 

According to ISO 20776-2, the modal MIC value determined with the test system may differ ± one doubling dilution step from the modal value obtained with the ISO 20776-1 method. At least 90% of the data obtained with the test system of interest need to be within this acceptable range (EA ≥ 90%). With these preconditions, at least 95% of the measured data must be reproducible. 

## 3. Results and Discussion

### 3.1. MIC Values Determined with Vacuum Dried Biocide Microtiter Plates Are in Agreement with Results Obtained with Broth Microdilution According to ISO 20776-1

**Figure 1:**
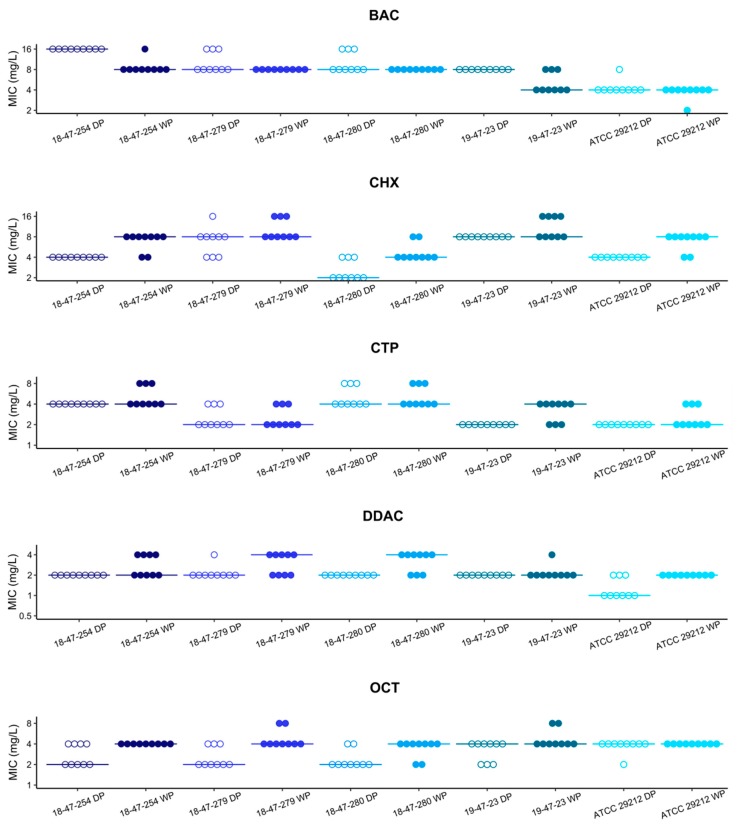
Susceptibility of three *Enterococcus faecium* (18-47-254, 18-47-279, 18-47-280) and two *E. faecalis* (19-47-23, ATCC 29212) strains to cationic biocides. Minimum inhibitory concentrations of benzalkonium chloride (BAC), chlorhexidine dihydrochloride (CHX), cetylpyridinium chloride (CTP), didecyldimethylammonium chloride (DDAC), and octenidine dihydrochloride (OCT) determined by wet plate procedure (WP; filled dots) and dried plates (DP; empty dots) are shown. Lines represent the respective modal values.

**Figure 2:**
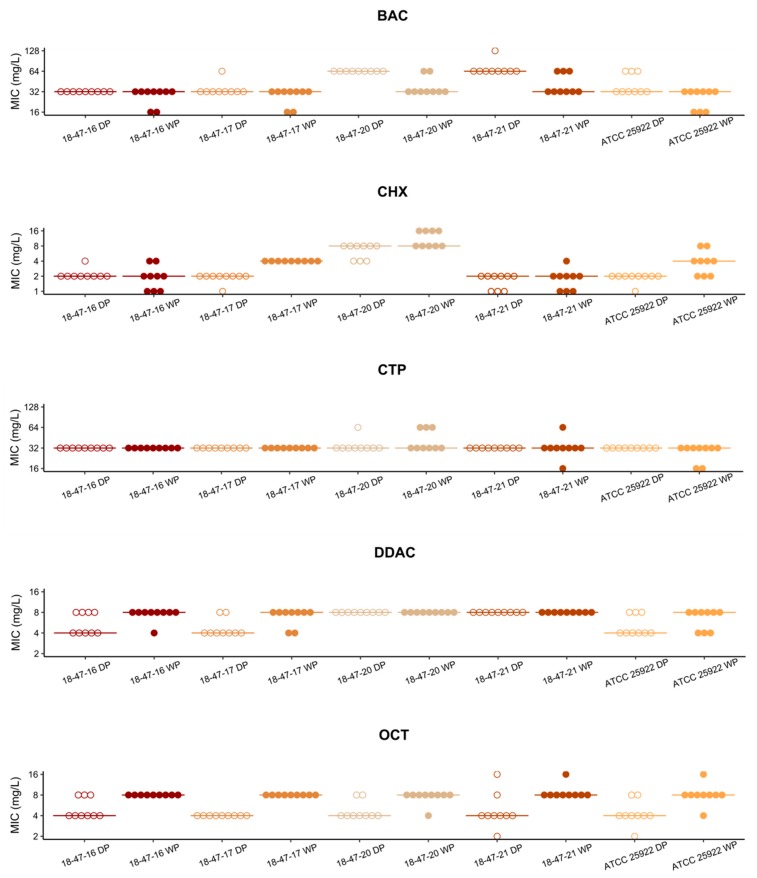
Susceptibility of five *Escherichia coli* strains to cationic biocides. Minimum inhibitory concentrations of benzalkonium chloride (BAC), chlorhexidine dihydrochloride (CHX), cetylpyridinium chloride (CTP), didecyldimethylammonium chloride (DDAC), and octenidine dihydrochloride (OCT) determined by wet plate procedure (WP; filled dots) and dried plates (DP; empty dots) are shown. Lines represent the respective modal values.

In our study, MIC values were determined using two independent methods for an initial panel of three *E. faecium*, two *E. faecalis* (Figure 1), and five *E. coli* strains (Figure 2). The MIC values determined with the customized vacuum dried biocide microtiter plate were within the acceptable range of ± one doubling dilution step compared to the modal values obtained with a broth microdilution method according to ISO 20776-1 (EA = 100% for all isolate-substance combinations). For enterococci, data obtained from all replicates were within the acceptable range of ± one doubling dilution step (reproducibility = 100% for all measurements). For *E. coli*, reproducibility reached 100% in CTP and DDAC. It was lower in BAC (98%), CHX (96%) and OCT (96%) but was still within an acceptable range (≥95%) according to ISO 20776-2. 

Taken together, vacuum dried biocide microtiter plates provide a performance level comparable with broth microdilution (the ISO 20776-1 reference method). Hence, the evaluated microtiter plates are suitable for quick and standardized MIC testing of cationic biocides. Broad MIC ranges allowed for susceptibility testing of gram-positive and gram-negative species with the same plate design. However, it needs to be noted that this method is restricted to biocidal substances that can be easily de- and rehydrated such as the cationic biocides tested in our study. Customized vacuum dried biocide microtiter plates have been used for biocide susceptibility testing in three independent studies before [18,19,20]. The microtiter plates were also manufactured by MERLIN Diagnostika GmbH and all contained acriflavine, alkyldiaminoethyl glycin hydrochloride, benzethonium chloride, BAC, and CHX as heavy metal salts. However, none of these studies reported on the comparability of results with data obtained by broth microdilution according to ISO 20776-1.

### 3.2. Cationic Biocide Susceptibility Profiles of Vancomycin Resistant and Susceptible E. faecium Are Similar

The vacuum dried biocide microtiter plate system was used to determine the susceptibility of 90 *E. faecium* strains to cationic biocides (Table 1). Reproducibility reached 100% for each substance by accepting a variability of ± one doubling dilution step. The high reproducibility points towards the reliable description of susceptibility patterns for cationic biocides by applying a test system based on vacuum dried plates. Overall, MIC and MBC values of BAC, DDAC, and CHX were in accordance with previously reported data on *E. faecium*, which were generated by broth microdilution according to ISO 20776-1 [13,21], modified broth microdilution and subsequent macrodilution [22,23], or agar dilution [14]. Interestingly, compared to data published by Morrissey et al. [21], MIC values of CHX were quite low (2–4 mg/L vs. 16 mg/L) in our subpopulation of *E. faecium* isolates, which might display geographic variability. Unfortunately, data on susceptibility to CTP and OCT are not available so far. MIC_95_ and MBC_95_ values of OCT, CTP, and DDAC in VRE and VSE were concordant. The MBC_95_ of BAC and the MIC_95_ of CHX were twice as high in VSE compared to VRE. This observation is in contrast to previous findings, where vancomycin resistance was associated with reduced susceptibility to cationic biocides, such as BAC and CHX [13,14]. While our study focused on clinical *E. faecium* isolates from wards with low CHX usage, the study conducted by Alotaibi et al. investigated isolates from Danish hospital wards, where CHX is heavily used [13], which might be one reason for the observed difference in study outcomes. Last but not least, BAC and CHX susceptibility of VRE and VSE differed only in one doubling dilution step, which is within the acceptable range in terms of comparability of results according to ISO 20776-2. Mechanisms mediating reduced biocide susceptibility in enterococci are still not well understood. However, in some studies reduced susceptibility to substances such as CHX and BAC was linked to increased efflux pump activities [13,24,25], which is in line with common biocide tolerance mechanisms described for various bacterial species [26]. Although increased efflux pump activity can be associated with resistance to certain antibiotics in enterococci, e.g., streptogramins, tetracyclines and quinolones [27], there is no evidence that efflux pumps contribute to vancomycin resistance. In enterococci, identified vancomycin resistance mechanisms include target modification and removal of high affinity precursors that are usually synthesized in the cell. Both mechanisms result in reduced binding of vancomycin to the bacterial cell [27,28]. It has been shown that adaptation to biocides can result in modification of bacterial cells. The adaptation of *K. pneumoniae* to CHX, for example, was linked to the upregulation of genes involved in modification of the outer membrane [29]. Whether adaptation to cationic biocides like CHX leads to an alteration of the enterococcal cell wall, which might consequently affect the binding of vancomycin, needs to be investigated in future studies. Results of our study do not provide evidence of an association between reduced susceptibility to cationic biocides and vancomycin resistance.

## 4. Conclusions

In our study, we explored the suitability of a newly developed biocide susceptibility test system based on microtiter plates containing vacuum dried biocides as a screening method to identify bacteria resistant to cationic substances frequently used in hospital settings. We were able to show that this test system provides reliable results similar to the broth microdilution method according to CLSI guidelines and ISO 20776-1. Based on the data collected, the test system is appropriate for both, gram-positive and gram-negative species and may, therefore, serve as a fast and easy-to-handle surveillance tool for biocide-tolerant bacterial isolates. In a clinical application trial, we determined the susceptibility of 90 clinical *E. faecium* isolates to cationic biocides. Our results revealed no association of biocide tolerance with vancomycin resistance in the strain collection under study. In summary, monitoring and early identification of clinical isolates tolerant towards disinfectants applied in hospitals will help to adjust hygiene measures and to control nosocomial infections while simultaneously reducing antibiotic consumption.

## Figures and Tables

**Table 1:** Susceptibility of *Enterococcus faecium* to cationic biocides tested by a customized microtiter plate (MERLIN Diagnostika GmbH).

Biocide (Concentration Range Tested)	Species	Number of Isolates with MIC Value (mg/L) of							MIC_95_	Number of Isolates with MBC Value (mg/L) of							MBC_95_
		0.25	0.5	1	2	4	8	16		0.25	0.5	1	2	4	8	16	
BAC (0.5–256 mg/L)	VSE			1		11	36		8						41	7	16
	VRE					15	27		8						42		8
CHX (0.25–128 mg/L)	VSE		1	2	35	10			4				2	1		45	16
	VRE			1	41				2				3	1	3	35	16
CTP (1–256 mg/L)	VSE			3 *	17	28			4					48			4
	VRE				11	31			4					42			4
DDAC (0.5–128 mg/L)	VSE		1 ^#^	25	22				2				48				2
	VRE			26	16				2			2	40				2
OCT (0.125–32 mg/L)	VSE		1	8	39				2				22	26			4
	VRE			4	38				2				22	20			4

Biocide concentrations which have not been tested are shaded in gray. BAC = benzalkonium chloride, CHX = chlorhexidine dihydrochloride, CTP = cetylpyridinium chloride, DDAC = didecyldimethylammonium chloride, OCT = octenidine dihydrochloride, VRE = vancomycin resistant *Enterococcus faecium*, VSE = vancomycin susceptible *Enterococcus faecium*, ^#^ MIC ≤ 0.5, * MIC ≤ 1.

## Supplementary Materials

The following are available online at https://www.mdpi.com/2076-2607/8/4/551/s1, Table S1: *E. faecium* study population.
